# Contrasting characteristics of psychosis in outpatients with borderline personality disorder or schizophrenia at a tertiary care institution

**DOI:** 10.3389/fpsyt.2024.1485000

**Published:** 2024-12-11

**Authors:** Mario Hernández-Velázquez, Adriana Díaz-Anzaldúa, Iván Arango, Mauricio Rosel-Vales, César Celada-Borja

**Affiliations:** ^1^ Department of Education Ramón de la Fuente Muñiz National Institute of Psychiatry, Mexico City, Mexico; ^2^ Department of Genetics, Sub directorate of Clinical Research, Ramón de la Fuente Muñiz National Institute of Psychiatry, Mexico City, Mexico; ^3^ Borderline Personality Disorder Clinic, Directorate of Clinical Services, Ramón de la Fuente Muñiz National Institute of Psychiatry, Mexico City, Mexico; ^4^ Schizophrenia Clinic, Directorate of Clinical Services, Ramón de la Fuente Muñiz National Institute of Psychiatry, Mexico City, Mexico

**Keywords:** mental health, tertiary care, borderline personality disorder, schizophrenia, psychosis, hallucination

## Abstract

**Summary and objectives:**

Borderline personality disorder (BPD) and schizophrenia can present with psychotic symptoms, such as delusions and hallucinations. This study, conducted at a tertiary care center, compared the characteristics of psychotic symptoms in patients diagnosed with BPD and patients diagnosed with schizophrenia, as well as the prevalence of self-harm, suicide attempts, and hospitalizations within these groups.

**Method:**

In this comparative study, 50 individuals diagnosed with BPD and 50 with Schizophrenia, aged between 18 and 45 years, were assessed for intensity of psychotic symptoms with the Psychotic Symptom Assessment Scale (PSYRATS) and the Cardiff Abnormal Perceptions Scale (CAPS). Data were analyzed with IBM SPSS v25.0.

**Results:**

On the PSYRATS, the schizophrenia group scored higher in auditory hallucinations and in the number of voices, while in the BPD group the auditory hallucinations score was correlated with the number of suicide attempts (P=0.025). On the CAPS, the BPD group showed higher scores on positive abnormal perceptions in all dimensions compared to the schizophrenia group (P=0.002).

**Conclusions:**

Our study suggests that patients with BPD experienced a more intense burden of psychotic-like experiences compared to those with Schizophrenia, with a greater frequency, interference, and distress reported. Although patients with Schizophrenia had higher scores on the PSYRATS, the BPD group’s scores were also notable, and a correlation was identified between auditory hallucinations and suicide attempts in the BPD group.

## 1 Introduction

Borderline Personality Disorder (BPD) and schizophrenia are serious mental disorders that profoundly affect emotional regulation along with social and occupational functioning. The former is characterized by a generalized dysfunction of the emotional regulation system, manifested as identity alterations, unstable and intense interpersonal relationships, impulsive behavior, self-harm, uncontrolled anger, affective instability, intolerance of aloneness, and a chronic feeling of emptiness (1). The latter is a complex syndrome that includes positive, cognitive and negative symptoms, causing a significant disruption in the social life of individuals (2).

Psychotic symptoms, which manifest as delusions and hallucinations, can be present in both disorders (3). Traditionally, hallucinations have been considered indicative of an underlying psychotic disorder, although recent evidence suggests their prevalence is as high as 25 to 30% in the general population, without a diagnosis of psychosis (4).

Research in this field has advanced significantly, identifying biopsychosocial risk factors and developing effective treatments. However, a gap remains in the understanding of psychotic symptoms in these disorders, especially when comparing BPD and schizophrenia with respect to the main characteristics of these symptoms, such as frequency, interference with activities, and distress generated by them (5).

Various studies have documented the prevalence and characteristics of psychotic symptoms in BPD and schizophrenia (6–10) and propose that hallucinations are not only common in schizophrenia but also in BPD, in this case being associated with a greater number of comorbid diagnoses, hospitalizations, self-harming behaviors, and suicidal attempts (5). Recent literature has noted that auditory hallucinations in patients with BPD are comparable in intensity and frequency to those experienced by patients with schizophrenia. However, distress and impact on daily life appear to be greater in BPD (11). Hallucinations in BPD also occur in different modalities, with a prevalence of 21 to 59% for auditory, 30 to 33% visual, 10 to 30% olfactory, and 13% tactile hallucinations (12).

The designation of hallucinations in BPD as pseudo-hallucinations has been subject to review, suggesting that terms such as quasi- or pseudo-hallucinations may not be the most appropriate to describe these experiences (12). Some studies confirm that these psychotic experiences have similar phenomenological characteristics to those of schizophrenia, which raises the need to review current diagnostic and therapeutic models (6, 13). Other studies have shown that psychotic-like experiences in patients with BPD are comparable to those of schizophrenia, both in the phenomenology of auditory hallucinations and in the distress they generate (10). However, the nomenclature used to describe hallucinations in BPD suggests that they are less severe and of shorter duration compared to those in schizophrenia (8).

The assessment of psychotic symptoms in these disorders is carried out using scales such as the Psychotic Symptom Rating Scale (PSYRATS) and the Cardiff Anomalous Perceptions Scale (CASP). The PSYRATS is a detailed tool for the evaluation of auditory hallucinations, which allows measuring various perceptual abnormalities and phenomenologically differentiating between them. Its version adapted to Spanish, composed of 11 items for hallucinations and 6 items for delusions, has proven to be a useful tool to reflect treatment results (14). For its part, CASP covers sensory and perceptual experiences not captured by other psychiatric tests, and allows the intensity, frequency and disturbance of each experience to be evaluated. Its validation in Colombia reflects educational and social characteristics typical of low- to middle-income countries, without finding significant differences by gender, educational level or marital status (15).

Although there is emerging evidence on the prevalence of psychotic symptoms in BPD, more research is required on their differentiation from psychotic symptoms in schizophrenia.

The aim of this study was to compare the characteristics of psychotic symptoms between patients diagnosed with BPD and schizophrenia using the PSYRATS and CAPS scales. Specifically, we sought to determine the intensity of psychotic symptoms in patients with those diagnoses in the collected sample, and to identifying the prevalence of self-harm, suicide attempts, and hospitalizations in the studied sample. We sought to contribute to a better understanding of psychotic symptoms in BPD, which is crucial for differential diagnosis and the development of more effective treatments.

## 2 Methods

### 2.1 Study design and sample

The current observational, cross-sectional and comparative study was carried out at the Ramón de la Fuente Muñiz National Institute of Psychiatry (INPRFM) in Mexico City. This design allowed the characteristics of two groups of patients (BPD and schizophrenia) to be simultaneously examined and compared at a specific point in time, which was suitable for exploring differences and similarities in their clinical and sociodemographic profiles without the need for a long-term follow-up. Based on an analysis conducted with G*Power v3.1.9.6 (16), a minimum sample size of 49 individuals per group was required for this study to achieve 80% power, and a 0.5 correlation, according to previous studies evaluating psychotic symptoms in individuals with BPD or comparing BPD with schizophrenia (17, 18). The final sample included 100 participants who had experienced psychotic symptoms: 50 diagnosed with BPD and 50 with schizophrenia, all aged between 18 and 45 years and diagnosed according to DSM-5 criteria. Exclusion criteria were substance use disorder (except for tobacco and caffeine), diagnosis of bipolar disorder type 1 and 2, post-traumatic stress disorder, intellectual disability or neurodevelopmental disorders, obsessive-compulsive disorder, major depressive disorder with psychotic symptoms, psychotic symptoms secondary to substances, hallucinations under the influence of substances, symptomatic severity that limited the application of the instruments, inability to basic understanding of the questions, difficulty communicating or cooperating, and other personality disorders. A non-random sampling was used. Participants who did not complete the assessments were eliminated from the study.

### 2.2 Measurements

The Psychotic Symptom Rating Scale (PSYRATS) was used, which consists of 11 items scored from 0 to 5, along with a 6-item delusion scale. Validated in Spain in 2003, it demonstrated high inter-rater reliability, with reliability coefficients above 0.85 across all 11 items (14). Additionally, the Cardiff Anomalous Perceptions Scale (CAPS) was applied. It is a 32-item questionnaire with Yes/No answers, which offers a total score from 0 to 32; each item evaluates three dimensions (degree of disturbance, interference with activities and frequency), using a Likert scale from 1 to 5. The scale was validated in Spain in 2014 and in Colombia in 2015, obtaining reliability scores greater than 0.70 in all its dimensions: 0.83 for the total score, 0.88 for anxiety, 0.87 for interference and 0.85 for the frequency (14).

### 2.3 Statistical analysis

The α level of 0.05 was adjusted for multiple testing, resulting in 0.0045. Descriptive and inferential analyses were carried out with the IBM SPSS v25.0 program. For quantitative variables, the Kolmogorov-Smirnov test was applied to define whether they followed a normal distribution. In its absence, medians were reported instead of means. Bivariate analyses were performed with Mann-Whitney U tests and Spearman correlation for quantitative variables without normal distribution and Chi square for nominal variables. For multivariate analysis, the Kruskal-Wallis test was used for quantitative variables without normal distribution.

### 2.4 Ethical considerations

Ethical principles were complied with in accordance with the regulations of the General Health Law on Health Research in Mexico, the CIOMS Guidelines, and the Declaration of Helsinki. The aim, characteristics of the study, potential benefits and risks were verbally explained to participants before they gave their written consent to participate. In addition, the two witnesses signed and confirmed the participant’s understanding of the procedures to be followed in this study. The study was carried out for one year, from October 2022 to November 2023, with data collection and clinical interviews on a single occasion.

## 3 Results

Of the 118 individuals invited to participate, 18 were excluded according to pre-established criteria. As shown in **Table 1**, individuals with BPD had a median age 11 years younger than patients with schizophrenia (P<0.001), and there was a greater proportion of women in the BPD group (P<0.00001). No significant differences were found in marital status or educational level between both groups (P= 0.89 and 0.62 respectively).

**Table 1:** Sociodemographic characteristics of the sample.

Age median (years)	Total N= 100		BDP N=50		Schizophrenia N=50		P value^a^
	28		24		35		
	n	(%)	n	(%)	n	(%)	
Sex							
Male Female	42 58	(42) (58)	8 42	(16) (84)	34 16	(68) (32)	<0.00001^b^
Marital status							
Single Married Divorced Consensual union	86 1 6 7	(86) (1) (6) (7)	43 0 2 5	(86) (0) (4) (10)	43 1 4 2	(86) (2) (8) (4)	0.89**^b^**
Educational level							
Primary Lower Secondary Upper Secondary Baccalaureate Graduate studies	4 28 50 15 3	(4) (28) (50) (15) (03)	2 8 32 7 1	(4) (16) (64) (14) (2)	2 20 18 8 2	(4) (40) (36) (16) (4)	0.62**^b^**

BDP, Borderline personality disorder. ^a^Mann-Whitney U test; ^b^χ^2^.

A higher percentage of individuales with BPD had a history of suicide attempts and self-harm (86% and 92%, respectively) compared to the schizophrenia group (28% for both variables, p< 0.00001). In the BPD group, there were more than three times as many participants with one or more suicide attempts compared to the schizophrenia group (p<0.00001). In the BPD group, six females had no suicide attempts, 11 had one or two attempts, and 25 had three or more. There was no difference between females and males, where one male had no attempts, two had one or two attempts, and five had at least three attempts (Fisher p=1). Also, no sex differences were found in the Schizophrenia group, where nine women had no attempts, five had one or two attempts, and two had at least three attempts vs. 27 men with no attempts, four with one or two attempts, and three with at least three attempts (Fisher p=0.18). While in the BPD group the majority (eight to nine out of ten people) had a history of more than five self-harm experiences, in the schizophrenia group the majority (seven out of ten) had no history of this type of experiences (p < 0.00001). There was no difference in the percentage of history of hospitalizations between the groups (p=0.86). Having been hospitalized once or twice was the most common situation in both groups, with 50% in the BPD group and 58% in the schizophrenia group (**Table 2**).

**Table 2:** Comparison of the frequency of clinical impact variables in borderline personality disorder (BPD) and the schizophrenia groups.

	BPD N=50				Schizophrenia N=50				P value (χ^2^)
Hospitalizations N (%)	0 15 (30)	1-2 25 (50)	≥3 10 (20)		0 14 (28)	1-2 29 (58)	≥3 7 (14)		0.86
Suicide attempts N (%)	0 7 (14)	1-2 13 (26)	≥3 30 (60)		0 36 (72)	1-2 9 (18)	≥3 5 (10)		<0.00001
Self-harm N (%)	0 4 (8)	1-2 1 (2)	3-5 2 (4)	>5 43 (86)	0 36 (72)	1-2 9 (18)	3-5 2 (4)	>5 3 (6)	<0.00001

**Figure 1:**
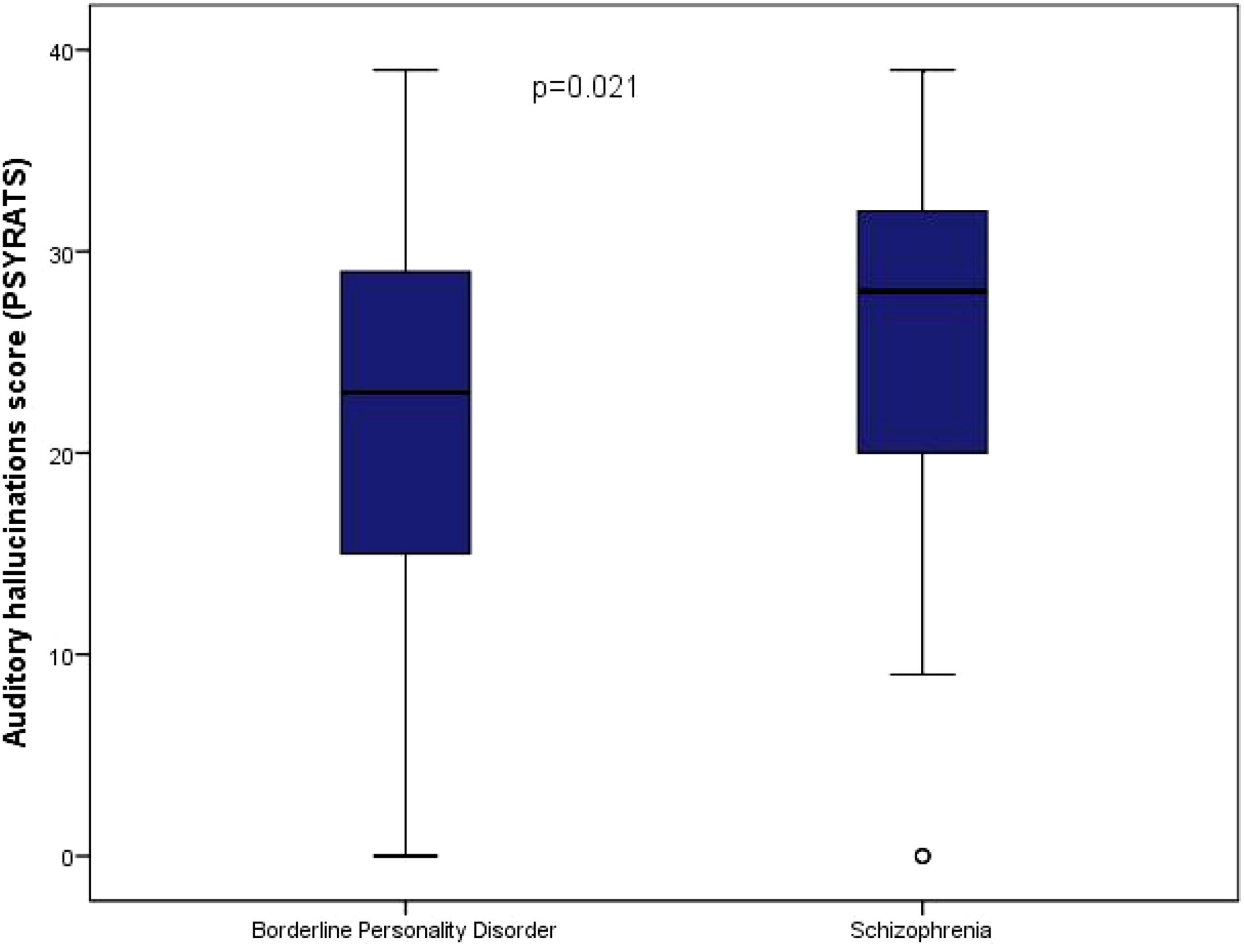
Auditory hallucinations according to the PSYRATS scale in both groups.

As shown in **Figure 1**, according to the PSYRATS scale, the median score for auditory hallucinations was 28 points in the schizophrenia group and 23 points in the BPD group (p = 0.021). The median number of voices was 4 in the schizophrenia group and 2.5 in the BPD group (P = 0.008). No differences were identified in the delusion score (p = 0.42), the duration of experiencing voices (p = 0.50), or delusions (p = 0.69) between the two groups.

**Figure 2:**
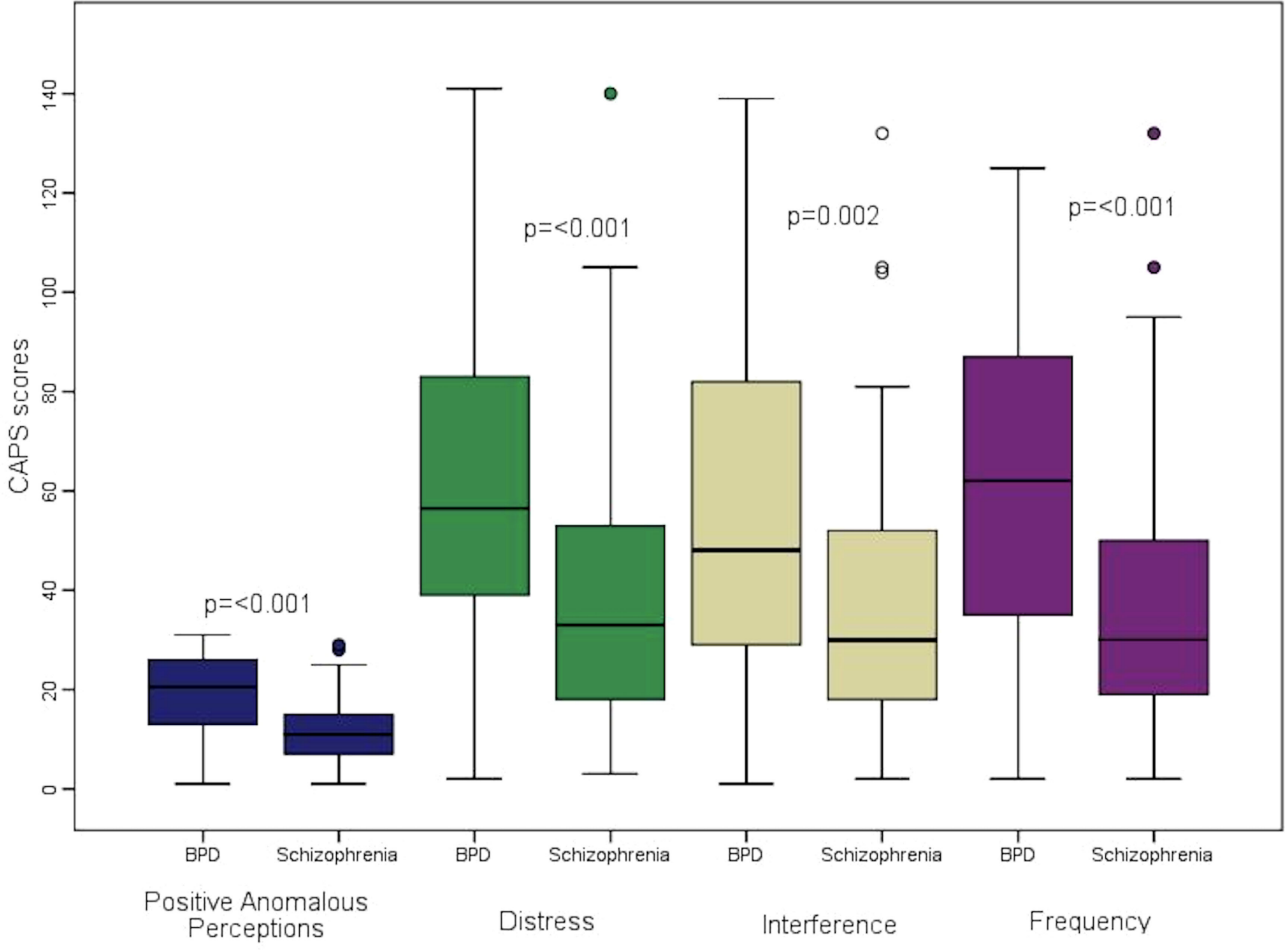
Comparison of CAPS clinical variables between the groups of borderline personality disorder (BDP) and schizophrenia.

As indicated in **Figure 2**, according to the CAPS scale, the median of total positive abnormal perceptions and the median of distress, interference, and frequency of positive abnormal perceptions were higher in the BPD group than in the schizophrenia group (p ≤ 0.002).

**Figure 3:**
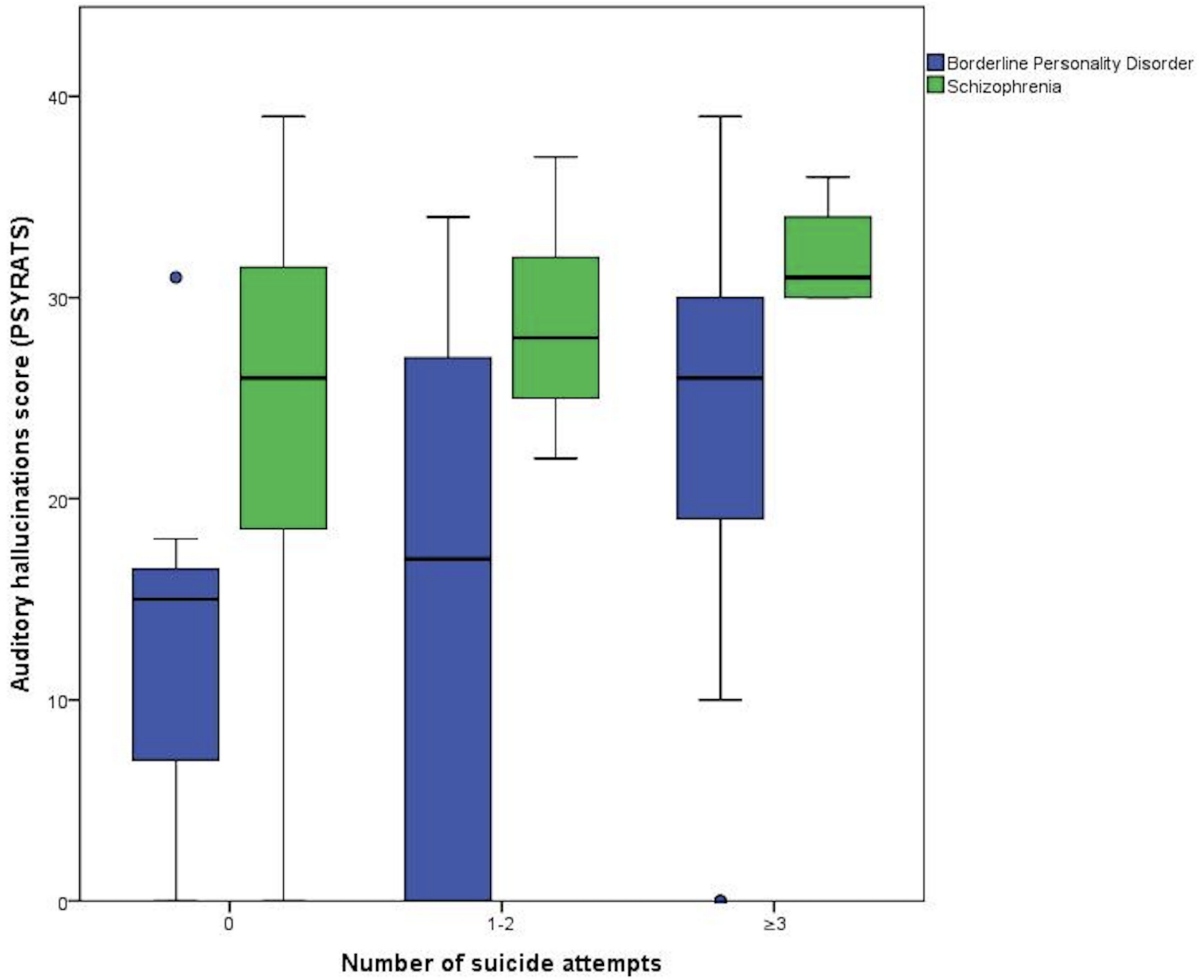
PSYRATS score according to number of suicide attempts in both groups.

**Figure 3** shows the auditory hallucinations score according to the suicide attempt categories assessed using the PSYRATS scale (p = 0.025, Kruskal-Wallis test). In the BPD group, the median auditory hallucinations score increased progressively, being lowest in individuals without a suicide attempt (15 points), followed by those with one or two attempts (17 points), and reaching its highest point in those with three or more attempts (26 points). In the schizophrenia group, progressive but not statistically different medians in the auditory hallucinations score were identified for those without suicide attempts (26 points), with one or two attempts (28 points) and with three or more attempts (31 points; P = 0.43, Kruskal-Wallis test).

No significant differences were detected in the hospitalization categories with respect to the auditory hallucinations score in the groups: BPD (p = 0.16) and schizophrenia (p = 0.86). There were no significant variations in the number of voices in various categories of self-harm, neither in the BPD group (p = 0.61) nor in the schizophrenia group (p = 0.64).

In the BPD group, no correlation was found between total positive anomalous perceptions, total distress score, interference, and frequency of anomalous perceptions with the frequency of hospitalizations, suicide attempts, or self-harm. However, in the schizophrenia group, a positive correlation was observed between suicide attempts and the total distress score and interference, respectively (**Table 3**).

**Table 3:** Spearman correlation (Rho) between the clinical impact variables and the Cardiff Anomalous Perceptions scale variables in both study groups.

	Anomalous perceptions		Distress		Interference		Frequency	
	ρ	P value	ρ	P value	ρ	P value	ρ	P value
BDP								
Hospitalizations	-0.08	0.576	-0.12	0.418	-0.10	0.500	-0.08	0.562
Suicide attempts	-0.01	0.939	0.06	0.701	0.07	0.625	0.07	0.622
Self-harm	0.17	0.240	0.25	0.082	0.27	0.060	0.22	0.121
Schizophrenia								
Hospitalizations	0.03	0.843	0.09	0.546	0.06	0.690	0.13	0.930
Suicide attempts	0.38	0.007	0.43	0.002	0.40	0.004	0.34	0.016
Self-harm	0.35	0.014	0.38	0.006	0.34	0.017	0.30	0.034

## 4 Discussion

Our comparative study between patients diagnosed with borderline personality disorder (BPD) and schizophrenia revealed that patients with BPD reported a more intense burden of psychotic experiences compared to patients with schizophrenia, despite being younger. BPD patients scored significantly higher on the CAPS scale, suggesting greater frequency, interference, and distress associated with these symptoms. While schizophrenia patients had higher scores for auditory hallucinations on the PSYRATS scale, the difference did not reach statistical significance under the adjusted threshold (p=0.0045). Both groups presented these symptoms, highlighting the importance of assessing psychotic experiences across disorders.

The findings in this study offer additional insights into the relationship between psychotic symptoms and clinical variables in patients with BPD and schizophrenia. Specifically, there were no significant differences in sociodemographic factors, such as marital status (p=0.89) and educational level (p=0.62), or in hospitalization rates (p=0.86), suggesting that these factors may not be crucial in the manifestation of psychotic symptoms in either group. Additionally, no differences were observed in the duration of hallucinations and delusions between the two disorders. The lack of correlation between auditory hallucination scores and self-harm across categories (BPD, p=0.61; schizophrenia, p=0.64) further suggests that self-harm may be driven by other factors beyond the frequency of these experiences. Similarly, the absence of significant differences in hospitalization rates based on auditory hallucination severity underscores that distress and coping mechanisms may play a more important role in determining hospitalization risk than symptom frequency alone.

These findings underscore the complexity of psychotic symptom interactions with clinical features in both BPD and schizophrenia, indicating that each disorder may require distinct therapeutic approaches (19). Notably, there were no significant differences in delusion scores on the PSYRATS scale between the two groups, showing the clinical relevance of addressing delusional ideation in BPD. Additionally, the comparable duration of auditory hallucinations between groups points to the importance of consistent monitoring and individualized treatment planning for patients with BPD (20, 21), as addressing the persistence and associated distress of these symptoms could improve outcomes. Together, these findings call for nuanced, symptom-focused interventions that account for the unique clinical profiles of patients with BPD and schizophrenia.

Auditory hallucinations in patients with BPD appear to have comparable intensity to those observed in schizophrenia, particularly in terms of distress and functional interference, aligning with previous research showing similar levels of suffering and impairment in both groups (17). This challenges the notion that hallucinations in BPD are inherently “less severe” (18). Notably, the higher prevalence and severity of self-harm behaviors in BPD suggest a unique link between impulsivity and psychotic symptoms in this disorder. Studies indicate that impulsivity and self-destructive behaviors are more common among younger individuals, potentially affecting comparisons across age groups (22). Additionally, BPD patients may experience hallucinations as a coping mechanism in response to trauma, rather than as a primary psychotic symptom, as proposed by literature on trauma-related voice hearing (23–26).

Moreover, these results are consistent with previous research documenting higher levels of distress and frequent hallucinations across sensory modalities in BPD. This study’s findings of a higher prevalence of self-harm and suicide attempts among BPD patients draw attention to the need for tailored interventions, as these behaviors are linked to the impulsivity often observed in younger individuals with BPD (22). Significant gender differences, such as the tendency of women to internalize symptoms through self-harm and men to exhibit more externalizing behaviors, further suggest that gender-sensitive treatments could enhance therapeutic outcomes (25). Furthermore, the similarity in the intensity of auditory hallucinations between BPD and schizophrenia, particularly regarding distress and interference, challenges previous assumptions that these hallucinations are inherently “less severe” in BPD (27). Further research to confirm these findings could refine diagnostic models and optimize treatment strategies, differentiating approaches that focus on hallucinations from those targeting self-harm to better serve the unique needs of each patient group.

These phenomenological insights suggest the need for therapies tailored to the specific experience of auditory hallucinations in BPD. Psychotherapeutic approaches such as CBT for voices (28), AVATAR Therapy (29), and Relating Therapy (30) offer promising options by addressing both the distress caused by hallucinations and the beliefs that sustain them (28–30). CBT for voices works by helping patients reframe distressing beliefs about their voices (28), while AVATAR Therapy provides a digital representation of these voices, allowing patients to engage with them directly and foster a sense of control (29). Relating Therapy further supports patients by encouraging a less reactive stance toward their voices, building resilience (30). These therapies provide collaborative, adaptive approaches that align with patients’ needs and experiences, offering a safer alternative to traditional antipsychotic medication (31).

Recognition of the presence and intensity of psychotic symptoms in patients with BPD is essential for mental health professionals, given that these symptoms can have a significant impact on quality of life, the risk of self-harming and suicidal behaviors in this group (24). Therefore, it is recommended to conduct more research that comparatively examines patients with BPD who present sensory-perceptual alterations and those who do not, with the aim of identifying possible associations with other clinical aspects and risk factors. Likewise, it is important to investigate how the gender or sex of the patients can influence the manifestation of these symptoms.

This study has several important limitations that should be considered when interpreting the results. First, the use of a non-probabilistic sampling method and a relatively small sample size limit the generalizability of the findings. Although the sample size was adequate for the statistical analyses performed, the lack of randomization introduces a potential selection bias, and a small sample size may reduce statistical power, making the findings more susceptible to individual variations and context-specific factors. These issues are particularly relevant when studying complex symptoms like psychosis, where personal histories, trauma, and familial mental health backgrounds may play significant roles in symptom expression (32).

Additionally, the cross-sectional and observational design of this study restricts the ability to draw causal inferences between variables, limiting the interpretation of observed correlations. This study design captures data at a single time point, which prevents examining how psychotic symptoms and self-harm behaviors might change or influence each other over time. In complex disorders such as BPD and schizophrenia, symptom interactions may shift dynamically, highlighting the need for longitudinal designs that could provide more insight into symptom evolution and causation. It is important to note that the schizophrenia group in our study consisted of outpatients who were in relatively stable conditions, as evidenced by their ability to attend regular follow-up appointments and participate in a structured study protocol. This stability may suggest a lower symptom burden compared to more acute cases, which potentially narrows the observed differences in symptom impact between BPD and schizophrenia. Furthermore, the lack of control for potential confounding variables, such as the use of antipsychotics, other medications, impulsivity and psychiatric comorbidities, may influence the expression and severity of psychotic symptoms, self-harm, and related behaviors. Consequently, while this study identifies associations between variables, these findings cannot be interpreted as causal relationships.

Cultural and contextual factors also present key limitations to the generalizability of these findings. Psychotic symptoms may be experienced, perceived, and expressed differently depending on cultural context, potentially influencing both the manifestation of symptoms and patients’ tendencies to seek treatment. Furthermore, individual differences, such as socioeconomic background and access to mental health resources, may have influenced the results but were not fully accounted for in this study. Expanding future research to include samples with greater cultural diversity and broader demographic representation would strengthen the applicability of these findings across different populations. Additionally, considering the impact of these contextual factors may help to clarify how symptoms vary across diverse settings, offering a more comprehensive understanding of psychotic symptoms in BPD and schizophrenia.

To address these limitations, future studies should consider implementing randomized sampling, larger and more culturally diverse samples, and longitudinal study designs. Incorporating a wider range of contextual variables, such as socioeconomic factors, cultural background, and trauma history, could help improve understanding of the nuanced ways in which psychotic symptoms manifest and interact with other clinical features across diagnostic groups.

## 5 Conclusion

Our comparative study between patients diagnosed with BPD and schizophrenia highlights that BPD patients experience a greater burden of psychotic symptoms than stable schizophrenia patients, particularly in terms of frequency, interference, and associated distress. Although schizophrenia patients scored higher for auditory hallucinations on the PSYRATS scale, these differences did not reach statistical significance under adjusted thresholds, indicating that both groups exhibit these symptoms with potentially comparable persistence. This study underscores the complexity of psychotic experiences across different disorders and the necessity of refined, symptom-focused approaches in their management. The findings have the potential to influence clinical practices and mental health policies, to improve care for patients with BPD and schizophrenia.

Given the limited research directly comparing psychotic symptoms in BPD and schizophrenia, further studies are encouraged to build upon these findings. Continued exploration in this area holds valuable potential for refining diagnostic models and developing tailored interventions, ultimately contributing meaningful advancements to the field of mental health.

## Data Availability

The raw data supporting the conclusions of this article will be made available by the authors, without undue reservation.
